# Properties of Nanostructured Hydroxyapatite Prepared by a Spray Drying Technique

**DOI:** 10.6028/jres.109.041

**Published:** 2004-12-01

**Authors:** Laurence C. Chow, Limin Sun, Bernard Hockey

**Affiliations:** American Dental Association Foundation, Paffenbarger Research Center, National Institute of Standards and Technology, Gaithersburg, MD 20899; National Institute of Standards and Technology, Gaithersburg, Maryland 20899

**Keywords:** bioactive materials, carbonated apatite, hydroxyapatite, nanostructured, spray drying

## Abstract

In previous studies nano sized hydroxyapatite (HA) particles were prepared by solgel or precipitation methods, in which the products were washed by aqueous or non-aqueous liquids to remove impurities or undesired components. The washing is know to modify the surfaces of the cystalline particles. This study evaluated properties of nano HA materials prepared by a spray drying method in which the HA product was not exposed to any liquid after its formation. The spray drying apparatus consisted of a nozzle that sprayed an acidic calcium phosphate solution in the form of a fine mist into a stream of filtered air flowing through a heated glass column. The water and volatile acid were evaporated by the time the mist reached the end of the column, and the fine particles were collected by an electrostatic precipitator. Powder x ray diffraction patterns suggested the material was amorphous, exhibiting a single broad peak at 30.5° 2*θ*. However, high resolution transmission electron microscopic analysis showed that the particles, some of which were 5 nm in size, exhibited well ordered HA lattice fringes. Small area diffraction patterns were indicative of HA. Fourier transfer infrared spectroscopy showed patterns of typical of HA with small amounts of HPO_4_^2−^. The thermodynamic solubility product of the nano HA was 3.3 × 10^−94^ compared to 1 × 10^−117^ for macro scale crystalline HA. These results showed that a spray drying technique can be used to prepare nanometer sized crystalline HA that have significantly different physicochemical properties than those of its bulk-scale counterpart.

## 1. Introduction

The mineral component of bone and teeth consists primarily of non-stoichiometric and highly substituted hydroxyapatite (HA) in poorly crystalline or nearly amorphous forms. The “impurity” components that are present at significant levels in biominerals include sodium, potassium, magnesium, and strontium substituting for calcium, carbonate for phosphate, and chloride and fluoride for hydroxyl ions [1]. Because HA is stable under *in vivo* conditions and is osteoconductive, synthetic HA has been widely used in hard tissue repair applications, such as implant coatings [2] and bone substitutes [1].

The apatite crystallites in human bone, enamel, dentin and cementum are all extremely small in size [3] and can be considered as nanostructured materials. Because HA is the prototype of biological apatites, which are in nano crystalline forms, extensive efforts have been made to produce synthetic nano HA materials. Methods that have been used for preparing nano HA include chemical precipitation [4,5,6,7], in some cases followed by spray drying [8,9,10] or hydrothermal treatment [5,11], sol-gel approach [12,13,14], microemulsion techniques [15,16,17,18], precipitation from complex solutions followed by microwave heating [19,20], wet chemical methods incorporating a freeze drying step [21], mechanochemical synthesis [22,23], and electrodeposition [24]. Additional studies reported synthesis of composites of nano HA and bioactive organic components including HA-collagen, HA-chondroitin sulfate or HA-chitosan using direct precipitation method [25,26,27,28], nano HA-polyamide using HA slurry and solution method [29,30], and Ca-deficient nano HA-high molecular weight poly (D,L-lactide) through a solvent-cast technique [31]. In the above methods, the nano HA materials were formed in a solution environment, and in most cases, the product was washed with water or other solvents to remove impurities or undesired components. Since washing of HA with water or other solutions are known to modify the surface properties of HA [32], exposure of the nano particles to additional solution environments is likely to result in significant interactions between particle surfaces and the solvent, leading to modifications of the surface properties and a reduction in the high reactivity innate to the small size, high surface area, nano particles. A new spray drying technique for the preparation of nano particles of HA is described. In this method, the nano particles would not be exposed to any solution environment and therefore would retain their original, highly reactive surfaces.

## 2. Materials and Methods

### 2.1 The Spray Drying Process

An important feature of the new spray drying process is that evaporation of the liquid would lead to in situ precipitation of HA that is essentially free of undesired components or impurities. This process requires that the solution being sprayed contains only calcium and phosphate ions and an acid component needed to solubilize the calcium phosphate compound. The acid must be sufficiently volatile so that it can be readily evaporated in the spray drying process, but to achieve this, the volatile acid must also be a weak acid such that no significant amounts of the acid anions, which are not volatile, may be present at the end of the evaporation process. Fortunately, precipitation of HA, resulting from evaporation of water in the spray drying process, would cause a decrease in solution pH. This, in turn, would cause the weak acid to become increasingly more undissociated and therefore readily evaporated. Theoretical considerations and experimental tests led to the conclusion that both carbonic and acetic acids are good candidates for this purpose. Thus, HA-saturated solutions to be used in the spray drying process were prepared by dissolving HA in a dilute acetic acid (17.5 mmol/L) solution or carbonic acid (266 mmol/L) solution.

**Fig. 1:**
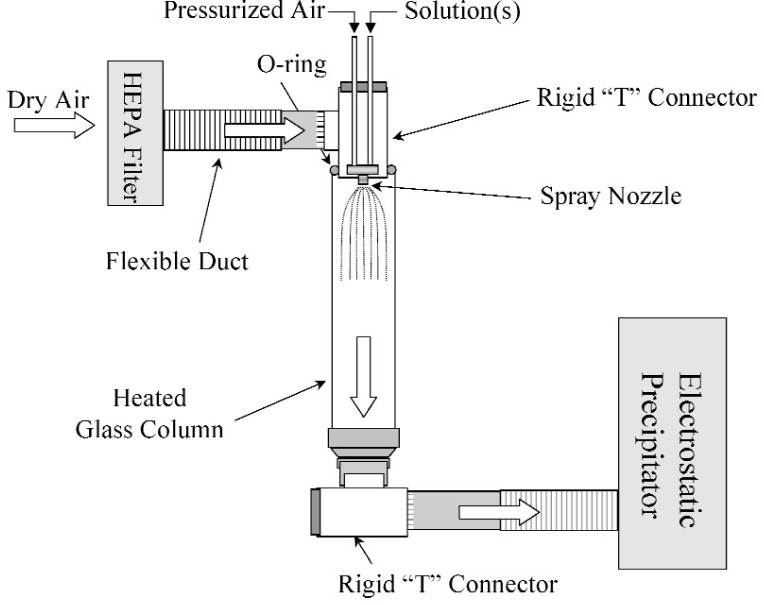
Schematic drawing of the spray drying apparatus.

The spray drying apparatus (Fig. 1) consisted of a spray nozzle^1^ (SUC1120, PNR America LLC, Poughkeepsie, NY) situated on the top of a glass column (Model VM770-48, VM Glass Co., Vineland, NJ, 6 in diameter), which was heated with electrical heating tapes (Model BIH 101100L, BH Thermal Co., Columbus, OH) and thermally insulted (fiberglass tape, Flextex, Montgomeryville, PA). HEPA filtered air was supplied at the top of the column, and an electrostatic precipitator (MistBuster, Air Quality Engineering, Inc., Minneapolis, MN) connected to the lower end of the column drew air from the column, creating a steady flow of air/mist through the column. The water and volatile weak acid in the solution were evaporated into the dry, heated air in the column and expelled from the precipitator into a hood. The fine particles suspended in the flow were trapped in the precipitator and collected at the end of the process.

### 2.2 Characterization of the Nano Particles

XRD (Rigaku DMAX 2200, Rigaku Denki Co., Ltd. The Woodlands, TX) was used to determine the crystalline phases [33] present in the product. Scans were performed between 10° < 2*θ* < 50°. The estimated standard uncertainty of the 2*θ* measurement is 0.01° and the mass fraction of a crystalline phase to be detected by XRD is about 3 %. It was anticipated that the product will contain primarily amorphous materials and the location and the intensity of the broad peak were noted.

A ThermoNicolet NEXUS 670 FT-IR spectrometer (Thermo Nicolet, Madison, WI) was used to record the infrared spectra of the nano powders. The powders were mixed with IR quality KBr at a mass ratio of ≈1:400 and finely ground in a mortar and pestle. The mixture was then pressed into a pellet in a 13 mm diameter evacuated die. The sample KBr pellet was run against the spectrum of a blank KBr pellet to cancel the impurity bands. The absorbance spectra were acquired over the range of 400 cm^−1^ – 4000 cm^−1^ using a DTGS detector and KBr beam splitter, with a resolution of 2 cm^−1^. Each spectrum was scanned 32 times to increase the signal-to-noise ratio. The estimated standard uncertainty of wavelength was ± 4 cm^−1^.

Multipoint Brunauer-Emmett-Teller (BET) surface area analyses were done (Gemini 2375 Surface Area Analyzer, Micromeritics, Norcross, GA) with ultra high purity nitrogen as the adsorbate gas and liquid nitrogen as the cryogen. The pressure sequence was (0.05, 0.10, 0.15, 0.20, 0.25) P/Po and the evacuation time was three min. The analysis mode was equilibration with the equilibration time of 5 s. The samples were dried in air overnight at 110 °C (Micromeritics Flow Prep station) before the measurement. Analyses were conducted on replicate samples to established standard deviation. In this and other measurements in the present study, the standard deviation was taken as the standard uncertainty.

A TA Q500 thermo gravimetric analyzer (TA Instruments—Waters LLC, New Castle, DE) was used to determine the weight loss of the nano powder sample with the increase of temperature. The temperature range was from 25 °C to 950 °C, and the heating rate was 10 °C/min. Estimated standard uncertainty of temperature calibration was ± 5 °C.

Samples of the nano materials were analyzed for calcium (Ca) and phosphate (P) by spectrophotometric methods [34] and carbon (C) by combusting the sample at 1000 °C in a constant oxygen flow and detecting the carbon dioxide by infrared absorption using a LECO CHN 2000 Analyzer (St. Joseph, MI) [35,36]. This information was used in conjunction with FTIR data to estimate the chemical composition of the nano samples.

Transmission electron microscopy (TEM) was used in characterizing the particles. For this purpose, particles were deposited onto Cu grids, which support a “holey” carbon film. The particles were deposited onto the support grids by deposition from a dilute suspension in acetone or ethanol. The particle shapes and sizes were characterized by diffraction (amplitude) contrast and, for crystalline materials, by high resolution (phase contrast) imaging. The characterization was primarily carried out using a JEOL 3010 high resolution electron microscope (JEOL, Peabody, MA), equipped with a Gatan Image Filter (with parallel EELS) and a light element EDS system.

It was anticipated that the nano HA materials would be more soluble than their macro scale counterpart. Thus, dissolution of a nano HA sample is likely to produce a solution that would be highly supersaturated with respect to the crystalline phase, leading to subsequent precipitation of the crystalline phase. The transient nature of the dissolution behavior was taken into consideration when conducting the solubility measurements as follows. The solubility experiments were conducted by dissolving the nano HA sample in solutions pre-saturated with crystalline HA at pH (5.0, 5.5, and 6.0). Based on calculations using a commercially available software “Chemist” (MicroMath, Salt Lake City, UT), the solutions were prepared by equilibrating crystalline HA in 8.1 mmol/L, 2.7 mmol/L, and 0.92 mmol/L phosphoric acid solutions, that also contained 150 mmol/L KNO_3_ as an electrolyte background, until saturation followed by filtration. In each solubility measurement conducted at (21 ± 1) °C, a pre-calibrated combination pH electrode [60110B, Extech Instruments Co., Waltham, MA] and a Ca-ion specific electrode [Orion 97-20 Ion Plus, Thermo Electron Co., Woburn, MA] were placed in 100 mL of a HA-saturated solution under constant stirring (52.4 rad/s or 500 rpm), and stable electrode readings, recorded on a computer, were obtained. A nano HA sample, 0.1 g in mass, was then added to the solution, and while the pH and Ca electrode readings were recorded every 10 s, 5 mL of the equilibrating slurry was removed at (1, 2, 3, 4, 5, and 10) min and immediately filtered for analysis of [Ca] and [P] concentrations using spectrophotometric methods [34]. The pH, [Ca], and [P] values were used to calculate solution ion activity products (IAP) with respect to HA [Eq. (1)] and other calcium phosphate phases using the software “Chemist”

$$\mathrm{IAP}(\mathrm{HA})={\left({\mathrm{Ca}}^{2+}\right)}^{10}{\left({\mathrm{PO}}_{4}\right)}^{6}{\left(\mathrm{OH}\right)}^{2}$$ (1)

where quantities in (·) on the right hand side of equation denote ion activities. Solubility measurements were conducted on replicate samples to established standard deviation.

## 3. Results

### 3.1 Properties of Acetic Acid-Derived Nano HA

**Fig. 3:**
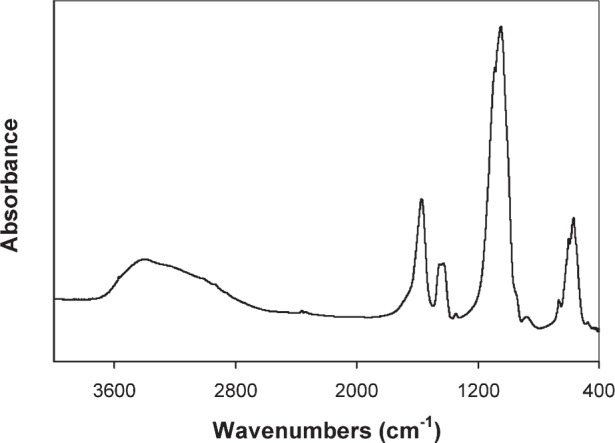
FTIR of nano HA prepared with acetic acid.

Once brushed off the precipitator plates, the nano HA had the form of a fine white powder. The sample exhibited XRD patterns typical of that from an amorphous material (Fig. 2a). TEM observations showed clusters that contained spherical particles about 10 nm to 100 nm in diameter (Fig 2b). High resolution TEM performed on particles that had been suspended in ethanol (95 % volume fraction) for 2 d showed packed crystalline HA particles 5 nm to 10 nm in size (Fig. 2c). Fourier transformed infrared (FTIR) analyses of the samples showed (Fig. 3) a pattern indicative of HA with the presence of some acid phosphate (874 cm^−1^, 1356 cm^−1^, 1389 cm^−1^), adsorbed water, and acetate (670 cm^−1^, 1417 cm^−1^, 1462 cm^−1^, 1568 cm^−1^). BET measurement results showed a surface area of (mean ± standard deviation, which is taken as standard uncertainty, *n* = 2) (33.1 ± 3.4) m^2^/g, leading to a calculated (assuming spherical particles) mean particle size of 58 nm. Elemental analysis showed that the materials had a carbon content of 5.79 % mass fraction (5.79 %) from acetate residue. Because calcium acetate is quite soluble and this may mask the true solubility of the nano HA, solubility measurements were not performed on this material.

### 3.2 Properties of Carbonic Acid-Derived Nano HA

**Fig. 4:**
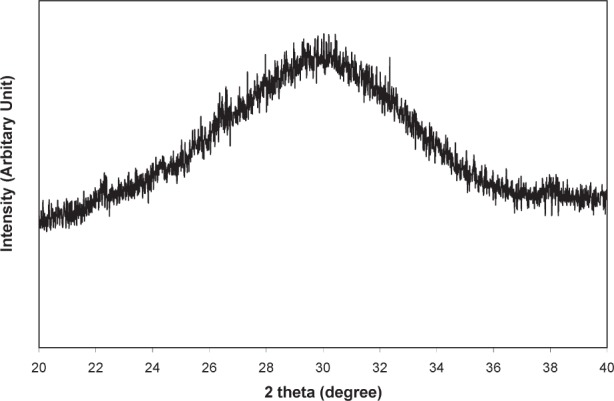
XRD of the nano HA prepared with carbonic acid.

**Fig. 5:**
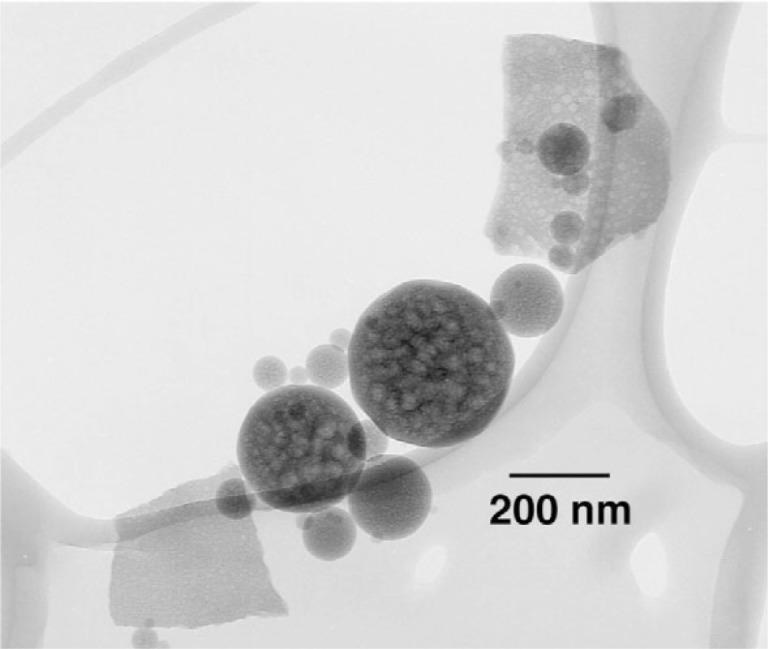
TEM of nano HA prepared with carbonic acid.

**Fig. 6:**
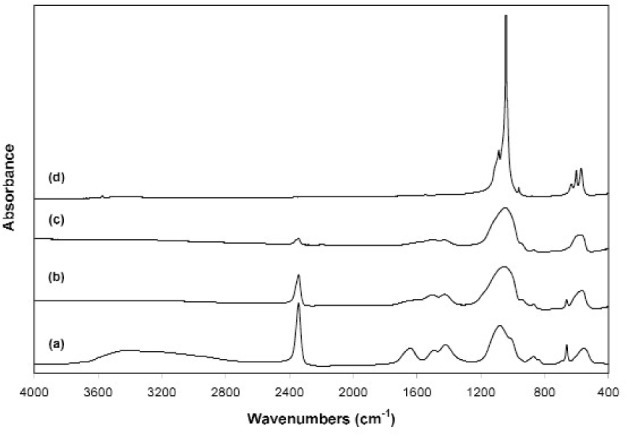
FTIR of the nano HA prepared with carbonic acid (a), showing effects of heating to 250 °C (b), 600 °C (c), and 950 °C (d).

The sample was a fine white powder and showed XRD patterns typical of an amorphous material (Fig. 4). TEM observations showed clusters of porous spherical amorphous particles that range from 50 nm to about 1 μm in size (Fig. 5). BET analysis showed surface area of (7.17 ± 0.19) m^2^/g (*n* = 2), leading to a calculated mean particle size of 266 nm. Because the material has the stoichiometry similar to that of HA but is amorphous under both XRD and TEM examinations, this material will be referred to as “amorphous HA” (AHA) in this paper. FTIR (Fig. 6a) showed the pattern of amorphous calcium phosphate with the presence of some acid phosphate (870 cm^−1^), adsorbed water (3407 cm^−1^), molecular water (1645 cm^−1^), and a large amount of trapped CO_2_ (2342 cm^−1^) as well as some carbonate incorporation in the structure (870 cm^−1^, 1422 cm^−1^, and 1499 cm^−1^) [37]. Elemental carbon analysis showed the material also contained 9.1 percent mass fraction (9.1 %) of carbon.

**Fig. 7:**
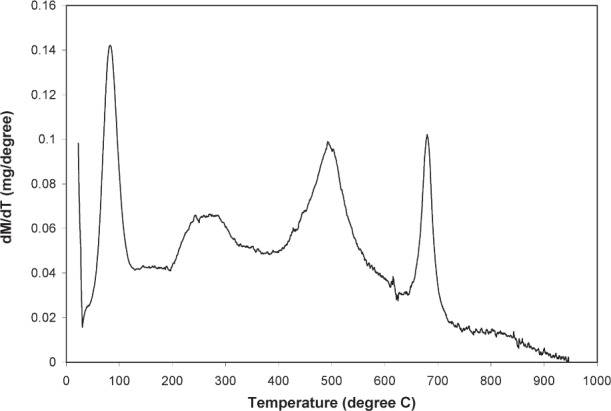
TGA of the nano HA prepared with carbonic acid showing weight losses at various temperatures.

Thermal gravimetric analysis (TGA) showed that sample mass losses occurred at (60 to120) °C, (210 to 380) °C, (440 to 580) °C, and (650 to 750) °C (Fig. 7). Most of the trapped CO_2_ was lost after being held for one hour in vacuum at 600 °C (Fig. 6c) and completely escaped after heating to 950 °C (Fig. 6d). The intensity of the carbonate bands in AHA (870 cm^−1^, 1422 cm^−1^, and 1499 cm^−1^) decreased with increasing temperature (Fig. 6a–c) and finally changed to type B (870 cm^−1^, 1457 cm^−1^, and 1552 cm^−1^) and type A (870 cm^−1^, 1457 cm^−1^, and 1421 cm^−1^) carbonate incorporation, substituting for phosphate and hydroxyl groups [38], respectively, as the AHA structure transformed to a carbonated HA after heating to 950 °C in vacuum (Fig. 6d).

**Fig. 8:**
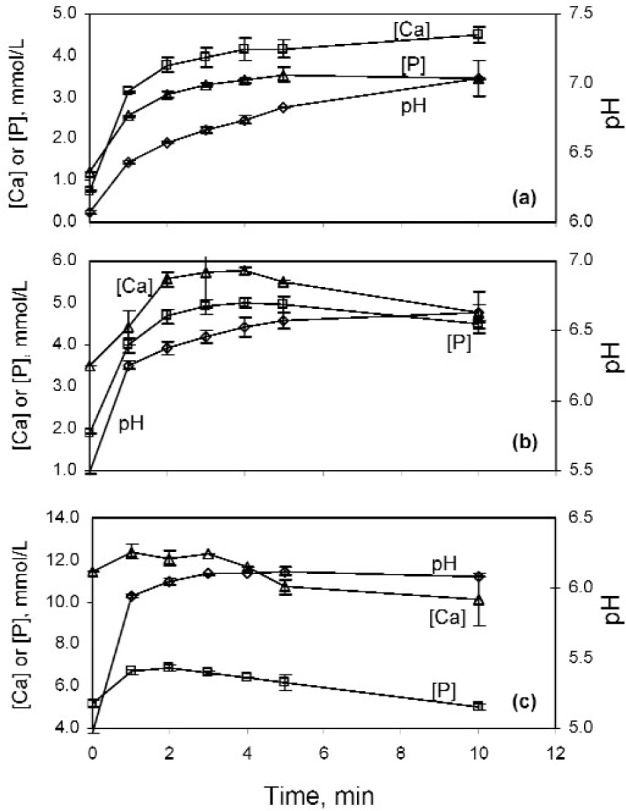
Changes in [Ca] and [P] concentrations and pH from dissolution of nano HA in a (a) pH 6, (b) pH 5.5, and (c) pH 5 HA-presaturated solutions.

The solubility results showed that in each dissolution experiment, the [Ca] and [P] concentrations as well as the pH increased rapidly with time (Figs. 8a to 8c). This indicated that the nano-HA was much more soluble than the crystalline HA. For dissolution experiments conducted with pH 5.0 and pH 5.5 HA-presaturated solutions, rapid increases in [Ca] and [P] were followed by gradual decreases in these concentrations starting at about 2 min (Figs. 8b and 8c), while the pH continued to increase. This observation suggested that a less soluble HA phase began to precipitate as the nano HA continued to dissolve. The calculated pIAP(HA) = −log [IAP(HA)] (see Eq. (1) for IAP definition) values were (mean ± standard deviation; *n* = 2) 99.7 ± 0.2, 97.2 ± 0.4, and 93.5 ± 0.3, respectively, for data obtained from dissolution experiments with HA-presaturated solutions having pH 5.0, 5.5, and 6.0. The smaller IAP values (more positive pIAP values), observed at the lower pHs probably was, in part, a result of the simultaneous dissolution-precipitation phenomenon referred to earlier.

## 4. Discussion

Both nano HA samples, prepared with acetic acid and carbonic acid, appeared amorphous in XRD (Figs. 2a and 4), but the former HA was crystalline as revealed by high resolution TEM (Fig. 2c) despite the extremely small particle sizes of 5 nm to 10 nm. It is noted that this sample for the high resolution TEM analysis was suspended in ethanol for 2 d and there is a possibility that a phase transformation may have occurred during this period. However, under similar sample handling conditions, the carbonic acid derived nano HA remained amorphous under TEM analysis. Because the acetic acid- and carbonic acid-HA solutions had identical [Ca] and [P] concentrations and the spray drying processing conditions were essentially the same, the differences in crystallinity of the nano HA samples prepared from the two solutions may be attributable to factors related to the nature of the acids.

It was anticipated that the nano HA prepared from carbonic acid would be more soluble than crystalline HA, both because of its small particle size and CO_2_ content. An IAP(HA) value as high as 3.3 × 10^−94^ (pIAP = 93.6), compared to 1 × 10^−117^ for crystalline HA, was obtained from experiments in which the nano HA was dissolved in the pH 6 HA-presaturated solution. In this dissolution run, the [Ca] concentration increased from the initial value of (0.75 ± 0.01) mmol/L in the crystalline HA-presaturated solution to a near plateau value of (4.5 ± 0.2) mmol/L at 10 min when the experiment ended. The [P] concentration similarly increased from the initial value of (1.2 ± 0.1) mmol/L to a stable value of (3.5 ± 0.2) mmol/L at 5 min. The pH of the solution continued to increase and reached 7.03 ± 0.01 at 10 min. Dissolution of the same nano HA into the pH 5 HA-presaturated solution led to initial increases in [Ca] and [P] concentrations as in the pH 6 experiment. However, the initial increases were followed by continued decreases in these concentrations beginning at about 2 min to levels that were below the starting [Ca] and [P] concentrations. These results suggested that addition of nano HA to a pH 5 HA-saturated solution led to sustained precipitation of crystalline HA. Such a process might be useful for remineralizing dental carious lesions or for occluding open dentinal tubules as a treatment for dental hypersensitivity.

As described above, by using a minimal amount of a volatile weak acid to prepare the spray drying solutions, the process is in theory capable of producing HA materials that contain little or no impurity components. In practice, a fair amount of acetate was found in the nano HA sample prepared with the acetic acid-HA saturated spray drying solution, and a large amount of trapped CO_2_ was present in the nano HA prepared with the carbonic acid-HA saturated solution. The amount of residual acid components in the spray dried product could be reduced by using a more dilute solution, i.e., with lower [Ca] and [P] concentrations, because a smaller amount of acid would be required to prepare the solution. A complication with HA preparation in general is that HA has a high “affinity” for carbonate. Carbonate is readily incorporated into the HA structure in conventional HA preparation processes unless scrupulous measures are taken to exclude CO_2_ from the system. Because HA is the most alkaline salt among all calcium phosphates that can be prepared in an aqueous system, a larger amount of acid is needed to prepare HA saturated solutions compared to saturated solutions of other calcium phosphates. Consequently, the residual acid problem is most pronounced in the HA preparation. In fact, preliminary data indicated that no residual acid was present in dicalcium phosphate dihydrate nano particles prepared by this process. These observations suggest that the spray drying technique should be useful for preparing nano particles of a range of calcium phosphate phases with minimum impurities.

## Figures and Tables

**Fig. 2a:**
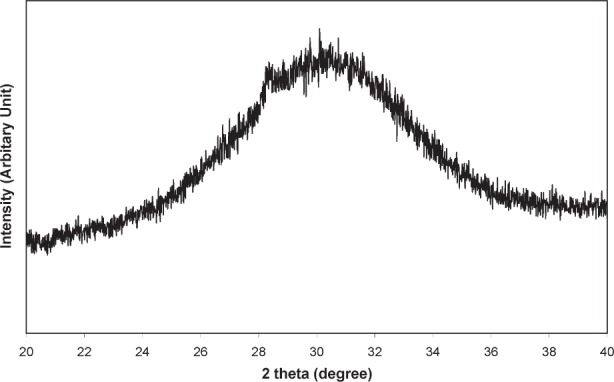
XRD of nano HA prepared with acetic acid.

**Fig. 2b:**
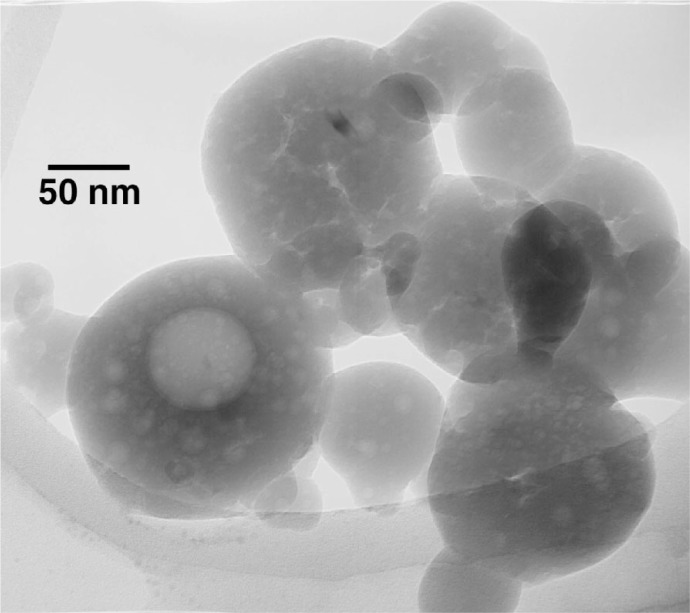
TEM of nano HA prepared with acetic acid.

**Fig. 2c:**
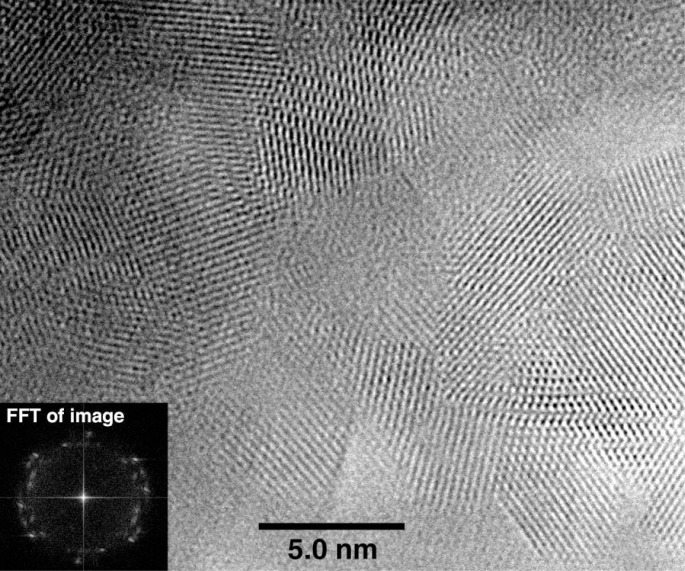
High resolution TEM of nano HA prepared with acetic acid.

## Footnotes

1 Certain commercial equipment, instruments, or materials are identified in this paper to foster understanding. Such identification does not imply recommendation or endorsement by the National Institute of Standards and Technology or the American Dental Association Foundation, nor does it imply that the materials or equipment identified are necessarily the best available for the purpose.
